# Lobomycosis: exuberant presentation with malignant transformation^☆^^☆☆^

**DOI:** 10.1016/j.abd.2021.05.004

**Published:** 2021-09-25

**Authors:** Wanessa da Costa Lima, Sidharta Quercia Gadelha, Mara Lúcia Gomes de Souza, Virginia Vilasboas Figueiras

**Affiliations:** Fundação de Dermatologia Tropical Heitor Vieira Dourado, Manaus, AM, Brazil

**Keywords:** Carcinoma, squamous cell, Lobomycosis, Mycosis fungoides, Skin ulcer

## Abstract

Lobomycosis is a chronic granulomatous infection caused by the yeast *Lacazia loboi*, typically found in tropical and subtropical geographical areas. Transmission occurs through traumatic inoculation into the skin, especially in exposed areas, of men who work in contact with the soil. Lesions are restricted to the skin and subcutaneous tissue, with a keloid-like appearance in most cases. The occurrence of squamous cell carcinoma on skin lesions with a long evolution is well known; however, there are scarce reports of lobomycosis that developed into squamous cell carcinoma. The authors report a patient from the Brazilian Amazon region, with lobomycosis and carcinomatous degeneration, with an unfavorable outcome, due to late diagnosis.

## Case report

A Brazilian indigenous male, 83 years old, farmer, from Santa Izabel do Rio Negro, state of Amazonas, Brazil, presented with disseminated skin lesions on his limbs for 30 years. He had a previous history of hypertension and worked with extractivism.

**Figure 1:**
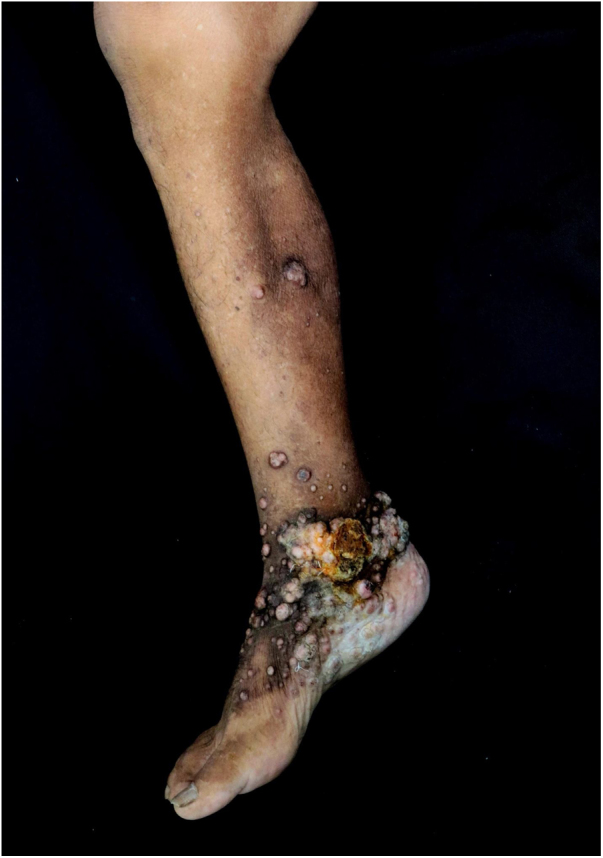
Keloid-like papules and nodules on the right lower limb.

**Figure 2:**
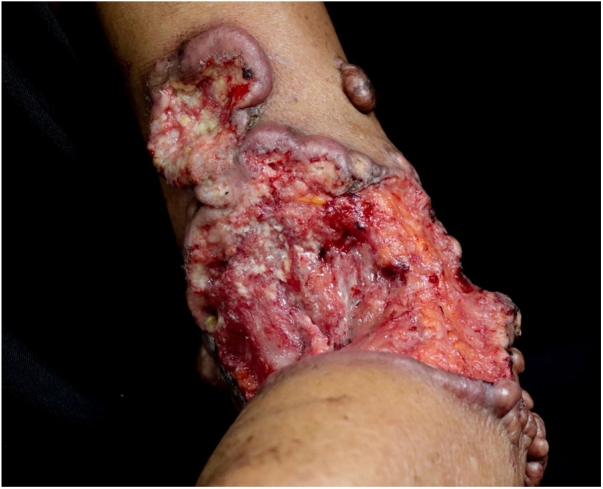
Ulcerated tumor, with exposed tendon, bleeding, raised edges, and presence of keloid-like nodules.

Upon physical examination, he had multiple keloid-like nodules on the upper and lower limbs. A vegetating lesion was seen on his right medial malleolus (Fig. 1) and the left cubital fossa showed an extensive ulcerated tumor with a four-month evolution, showing tendon exposure, persistent hemorrhage, and keloid-like edges (Fig. 2). He was initially treated with antibiotics in his municipality and later referred to the Dermatology service.

**Figure 3:**
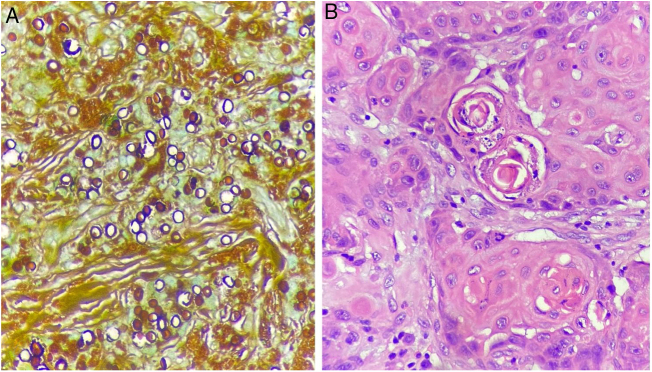
(A), Numerous fungal spores (Grocott, ×400). (B), Proliferation of tumor cells with the presence of atypical mitosis and horn pearls (Hematoxylin & eosin).

The histopathological examination of these two more recent lesions disclosed a thinned epidermis, hyalinization, and thickening of collagen fibers, in addition to the presence of multinucleated giant cells containing birefringent rounded fungal structures. Grocott staining, depicted these structures more clearly (Fig. 3A). Direct mycological examination showed elliptical, thick-walled fungal elements in a chain-like arrangement, isolated or presenting single budding.

Histopathology of the left upper limb tumor showed a dense lymphocytic inflammatory infiltrate, presence of horn pearls and proliferation of squamous cells with moderate pleomorphism, hypertrophic and hyperchromatic nuclei, establishing a diagnosis of squamous cell carcinoma (SCC) (Fig. 3B).

Immunohistochemistry revealed an infiltrating epithelial neoplasia with squamous differentiation and positivity for P63, CK5/6 and GATA3.

The oncological surgeon's opinion was requested, but the surgery for the excision of the SCC would be a large one and contraindicated in this patient due to his age, comorbidities and critical clinical condition. It was decided to implement palliative care and one month after hospital discharge, the patient died.

## Discussion

Lobomycosis, also called lacaziosis, keloid blastomycosis or Jorge Lobo's disease is a chronic granulomatous infection that can affect humans and dolphins. The etiological agent is the fungus *Lacazia loboi*, and its highest incidence is found in Central and South America, especially in the Amazon river basin.^1^, ^2^

Malignant transformation can occur on chronic ulcers, fistulas, and scars of different etiologies, including infectious ones, such as leprosy, tuberculosis, and lobomycosis. SCC is the most common skin cancer in these cases and results from the malignant proliferation of keratinocytes.^3^

**Figure 4:**
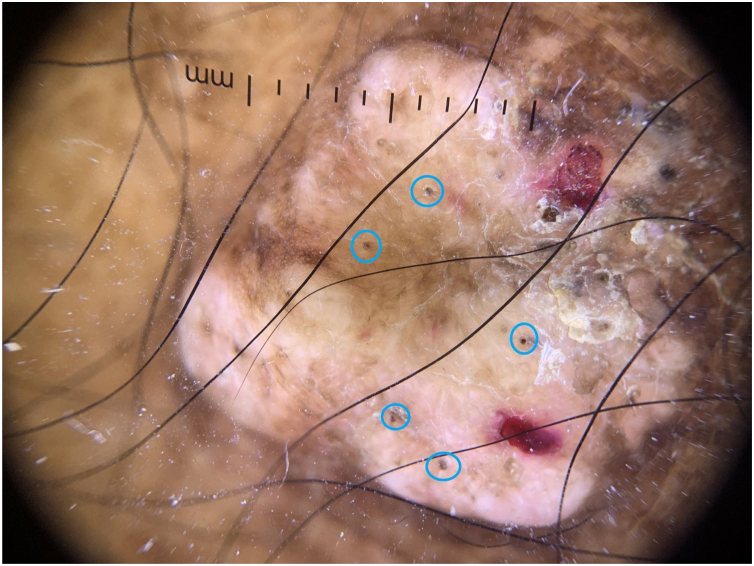
Dermoscopic image of a keloid-like nodular lesion on the lower limb (×10, non-polarized dermoscopy) Several black dots (blue circles).

There is no description in the literature of the dermoscopy of lobomycosis lesions. The findings of this case show the dermoscopy performed on a nodular lesion on the leg of this patient, with areas of hyperpigmentation interspersed with hypopigmentation, scales, blood crusts, and several black dots. Vascular structures were not observed (Fig. 4).

The choice of treatment is defined by the clinical presentation of the disease. In unifocal and localized forms, the surgical procedure is the most indicated treatment, and it can be associated with oral medications such as clofazimine, dapsone, and itraconazole, aiming to avoid recurrence. The combination of itraconazole and cryotherapy may be helpful in patients with large, multifocal lesions.^4^, ^5^

Patients with long-term lobomycosis associated whith SCC are rare. An early diagnosis and careful follow-up of these patients are essential to prevent the progression of the lesions and carcinomatous degeneration.

## Financial support

None declared.

## Authors’ contributions

Wanessa da Costa Lima: Approval of the final version of the manuscript; design and planning of the study; drafting and editing of the manuscript; collection, analysis, and interpretation of data; effective participation in research orientation; intellectual participation in the propaedeutic and/or therapeutic conduct of the studied cases; critical review of the literature; critical review of the manuscript.

Sidharta Quercia Gadelha: Design and planning of the study; intellectual participation in the propaedeutic and/or therapeutic conduct of the studied cases; critical review of the literature, critical review of the manuscript.

Mara Lúcia Gomes de Souza: Approval of the final version of the manuscript; design and planning of the study; drafting and editing of the manuscript; collection, analysis, and interpretation of data; effective participation in research orientation; intellectual participation in the propaedeutic and/or therapeutic conduct of the studied cases; critical review of the literature; critical review of the manuscript.

Virginia Vilasboas Figueiras: Approval of the final version of the manuscript; design and planning of the study; drafting and editing of the manuscript; collection, analysis, and interpretation of data; effective participation in research orientation; intellectual participation in the propaedeutic and/or therapeutic conduct of the studied cases; critical review of the literature; critical review of the manuscript.

## Conflicts of interest

None declared.
